# Oncogenic mutations in IKKβ function through global changes induced by K63-linked ubiquitination and result in autocrine stimulation

**DOI:** 10.1371/journal.pone.0206014

**Published:** 2018-10-18

**Authors:** April N. Meyer, Leandro H. Gallo, Juyeon Ko, Guillermo Cardenas, Katelyn N. Nelson, Asma Siari, Alexandre R. Campos, Thomas C. Whisenant, Daniel J. Donoghue

**Affiliations:** 1 Department of Chemistry and Biochemistry, University of California San Diego, La Jolla, California, United States of America; 2 Université Joseph Fourier Grenoble, Grenoble, France; 3 Proteomics Facility, Sanford Burnham Prebys Medical Discovery Institute, La Jolla, California, United States of America; 4 Center for Computational Biology and Bioinformatics, University of California San Diego, La Jolla, California, United States of America; 5 Moores Cancer Center, University of California San Diego, La Jolla, California, United States of America; Johns Hopkins School of Medicine, UNITED STATES

## Abstract

Mutations at position K171 in the kinase activation loop of Inhibitor of κB kinase beta (IKKβ) occur in multiple myeloma, spleen marginal zone lymphoma and mantle cell lymphoma. Previously, we demonstrated that these result in constitutive kinase activation and stimulate Signal Transducer and Activator of Transcription 3 (STAT3). This work also identified K147 as a site of K63-linked regulatory ubiquitination required for activation of signaling pathways. We now present a more detailed analysis of ubiquitination sites together with a comprehensive examination of the signaling pathways activated by IKKβ K171E mutants. Downstream activation of STAT3 is dependent upon the activity of: UBE2N, the E2 ubiquitin ligase involved in K63-linked ubiquitination; TAK1 (MAP3K7), or TGFβ Activated Kinase, which forms a complex required for NFκB activation; JAK kinases, involved proximally in the phosphorylation of STAT transcription factors in response to inflammatory cytokines; and gp130, or IL-6 Receptor Subunit Beta which, upon binding IL-6 or other specific cytokines, undergoes homodimerization leading to activation of associated JAKs, resulting in STAT activation. We further demonstrate, using an IL-6-responsive cell line, that IKKβ K171E mutants stimulate the release of IL-6 activity into conditioned media. These results show that IKKβ K171E mutants trigger an autocrine loop in which IL-6 is secreted and binds to the IL-6 receptor complex gp130, resulting in JAK activation. Lastly, by examining the differential abundance of proteins associated with K63-only-ubiquitinated IKKβ K171E, proteomic analysis demonstrates the global activation of proliferative responses. As cancers harboring K171-mutated IKKβ are likely to also exhibit activated STAT3 and p44/42 MAPK (Erk1/2), this suggests the possibility of using MAPK (Erk1/2) and JAK inhibitors, or specific ubiquitination inhibitors. K63-linked ubiquitination occurs in other kinases at sites homologous to K147 in IKKβ, including K578 in BRAF V600E, which serves as an oncogenic driver in melanoma and other cancers.

## Introduction

Many mutations in effectors and regulators of the nuclear factor kappaB (NFκB) signaling pathway have been identified in multiple myeloma, contributing to disease onset and viability [1]. Mutations at position 171 in the kinase domain of Inhibitor of κB kinase beta (IKKβ) have been identified in patients diagnosed with multiple myeloma [2], spleen marginal zone lymphoma [3] and mantle cell lymphoma [4]. Previously, we demonstrated that mutations at position 171 within the kinase activation loop of IKKβ result in constitutive kinase activation and induce activation of Signal Transducer and Activator of Transcription 3 (STAT3). This work also identified K147 as a site of K63-linked regulatory ubiquitination required for activation of signaling pathways [5].

IKKβ is the master regulatory kinase that activates the NFκB inflammatory pathway via Ser/Thr phosphorylation of Inhibitor of κB (IκB) proteins, thus targeting IκB proteins for degradation leading to the release of NFκB for nuclear translocation. In response to inflammatory stimuli, Transforming Growth Factor-Beta-Activated Kinase 1 (TAK1) activates IKKβ by phosphorylating Ser177, which primes the autophosphorylation of Ser181 in IKKβ [6]. IKKβ contributes to survival, stemness, migration and proliferation of many cancers including prostate cancer [7] and diffuse large B-cell lymphoma [8]. Activation of STAT3 is induced by the binding of IL-6 to the IL-6 Receptor (IL-6R), which leads to dimerization of IL-6 Signal Transducer, or gp130. Upon dimerization of gp130 subunits, the constitutively bound Janus Kinases (JAKs) become activated and phosphorylate Tyr705 of cytosolic STAT3, which translocates into the nucleus [9].

In this work, we present a more comprehensive examination of the signaling pathways activated by IKKβ K171E mutants, including a detailed analysis of ubiquitination sites. Downstream activation of STAT3 in response to IKKβ K171E mutants is dependent upon the activity of: 1) UBE2N, the E2 ubiquitin ligase involved in K63-linked ubiquitination; 2) TAK1 (MAP3K7), or TGFβ Activated Kinase, which forms a complex required for NFκB activation; 3) JAK kinases, involved proximally in the phosphorylation of STAT transcription factors in response to inflammatory cytokines; 4) gp130, or IL-6 Receptor Subunit Beta which, upon binding IL-6 or other specific cytokines, undergoes homodimerization leading to activation of associated JAKs, resulting in STAT activation. We further demonstrate, using an IL-6-responsive cell line, that IKKβ K171E mutants stimulate release into conditioned media of IL-6 activity. Lastly, by examining the differential abundance of proteins associated with K63-only-ubiquitinated IKKβ K171E, proteomic analysis demonstrates the global activation of proliferative responses.

## Results

### Wild type and mutant forms of IKKβ examined

**Fig 1:**
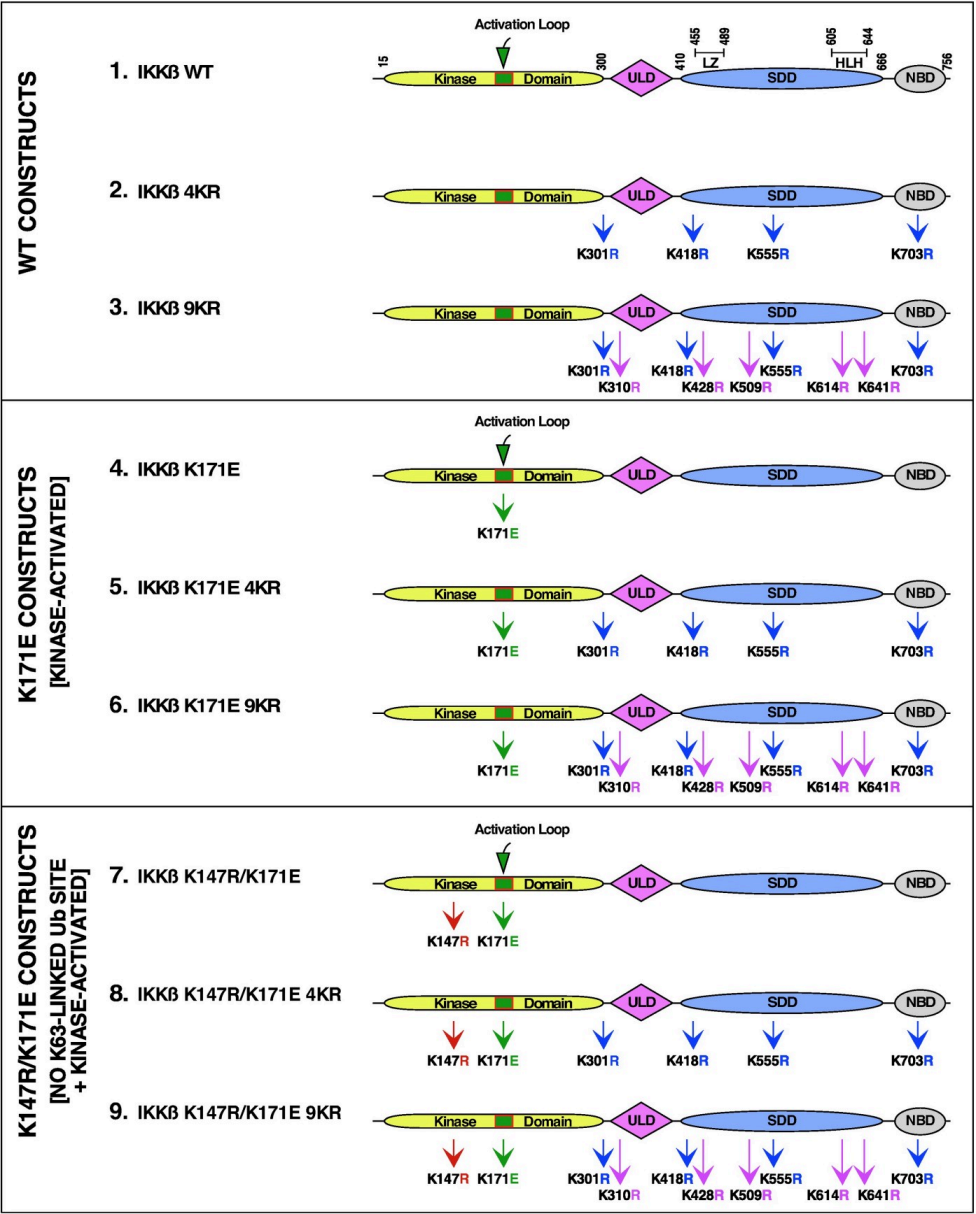
IKKβ constructs. (A) WT Constructs. Multiple sites of ubiquitination in IKKβ were identified by LC/MS-MS which were removed by site-directed mutagenesis. Initially, the IKKβ 4KR mutant was constructed with the mutations K301R, K418R, K555R, and K703R. Further analysis revealed additional ubiquitination sites, of which five more were mutated to create IKKβ 9KR with the additional mutations K310R, K428R, K509R, K614R, and K641R. (B) K171E Constructs. The kinase activating mutation K171E was introduced into the constructs shown in (A) to create IKKβ K171E, IKKβ K171E 4KR, and IKKβ K171E 9KR. (C) K147R/K171E Constructs. The mutation K147R, identified as a major site of K63-linked ubiquitination and required for IKKβ kinase activity [5], was introduced into the constructs shown in (B) to create IKKβ K147R/K171E, IKKβ K147R/K171E 4KR, and IKKβ K147R/K171E 9KR. The ubiqutin-like domain (ULD), the scaffold/dimerization domain (SDD) which contains the leucine zipper (LZ) and helix-looop-helix (HLH) regions, and NEMO binding domain (NBD) are indicated.

We previously utilized LC-MS/MS to show that IKKβ is ubiquitinated at K147, K301, K418, K555 and K703 and that among these, only K147 was required for the biological activity of the activated IKKβ [5, 10]. In the current work, additional novel sites of ubiquitination were identified by LC-MS/MS analysis: K310, K428, K509, K614 and K641, as discussed further below. The five newly identified sites were combined with four of the previously identified sites to create IKKβ 9KR. These proteins are introduced in Fig 1 (WT Constructs), which presents IKKβ WT, IKKβ 4KR, and IKKβ 9KR (Constructs 1, 2, 3).

Mutations at K171 within the kinase activation domain of IKKβ have been identified in several hemotological malignancies [2–4], either K171E or K171R. In this work, we have primarily examined the effects of the K171E mutation. This was combined with the mutations described above to create the proteins shown in Fig 1 (Middle: K171E Constructs, Kinase Activated), which presents IKKβ K171E, IKKβ K171E 4KR, and IKKβ K171E 9KR (Constructs 4, 5, 6).

Lastly, these mutations were combined with a mutation at the previously identified major site of K63-linked ubiquitination in IKKβ [5], the mutation K147R, which lies in the highly conserved motif HRDLK147 between the β6 and β7 domains of the N-terminal lobe of the kinase [11, 12]. This created the proteins shown in Fig 1 (Bottom: K147R/K171E Constructs, No K63-linked Ub site + Kinase Activated), which presents IKKβ K147R/K171E, IKKβ K147R/K171E 4KR, and IKKβ K147R/K171E 9KR (**Constructs 7, 8, 9)**. The mutation K147R significantly reduces the kinase activity.

### Activity of wild type and mutant forms of IKKβ

**Fig 2:**
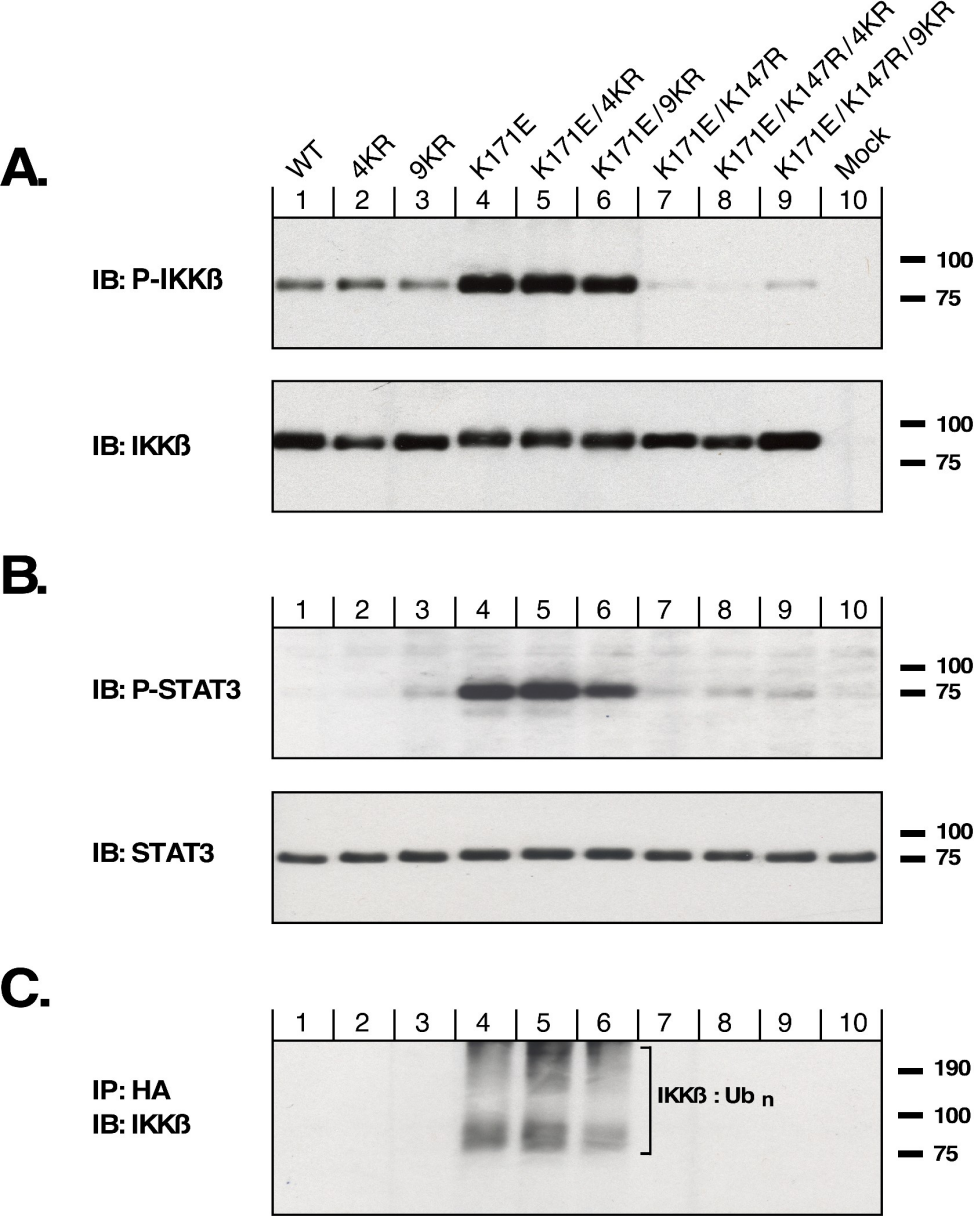
Phosphorylation and ubiquitination of IKKβ. HEK293T cells were transfected with the IKKβ mutants shown in Fig 1, in the same order, together with HA-Ub_3_. Cells were lysed in RIPA and proteins separated by SDS-PAGE. (A) Lysates were examined for activation of IKKβ kinase activity by immunoblotting for phospho-S177/S181 IKKβ. Total IKKβ expression is shown below. (B) The same lysates as in (A) were examined for STAT3 signaling by immunoblotting for phospho-Y705-STAT3. Total STAT3 is shown below. (C) HA-tagged ubiquitinated proteins from the same lysates were collected by immunoprecipitation, and HA-Ub-IKKβ was detected by immunoblotting for IKKβ.

This collection of IKKβ constructs was examined for activation of IKKβ using an antiserum that detects phosphorylation of IKKβ within the activation loop (Fig 2A). Although the proteins IKKβ WT, IKKβ 4KR, and IKKβ 9KR all exhibit activation (Lanes 1, 2, 3), introduction of the K171E mutation strongly increases activation as indicated by phospho-IKKβ immunoblotting (Lanes 4, 5, 6). In contrast, introduction of the kinase-dead mutation, K147R, essentially abolishes the phospho-IKKβ signal (Lanes 7, 8, 9).

We have previously utilized the phosphorylation of the signal transducing protein STAT3 to measure downstream signaling by activated IKKβ [5]. As shown in Fig 2B, the IKKβ mutants with the activating mutation K171E all exhibited strong activation of STAT3 signaling as shown by phospho-STAT3 (Lanes 4, 5, 6). In either the WT background (Lanes 1, 2, 3), or when combined with the kinase-dead mutation K147R (Lanes 7, 8, 9), significant phospho-STAT3 was not observed.

When this panel of IKKβ mutants was examined for total ubiquitination, using coexpression with an HA-tagged ubiquitin to allow anti-HA immunoprecipitation, immunoblotting to detect ubiquitinated IKKβ (Fig 2C) suggests that strong ubiquitination was observed only in the presence of the activating mutation K171E (Lanes 4, 5, 6). Having removed 4 ubiquitination sites in the IKKβ K171E 4KR protein (Lane 5), or 9 ubiquitination sites in the IKKβ K171E 9KR protein (Lane 6), we were initially surprised to see such significant ubiquitination in these proteins. This will be discussed further below. However, as expected, significant ubiquitination was not observed either for IKKβ proteins in the WT background (Lanes 1, 2, 3), nor for mutants with the kinase-dead K147R mutation (Lanes 7, 8, 9).

### Identification of major ubiquitination sites in IKKβ

**Fig 3:**
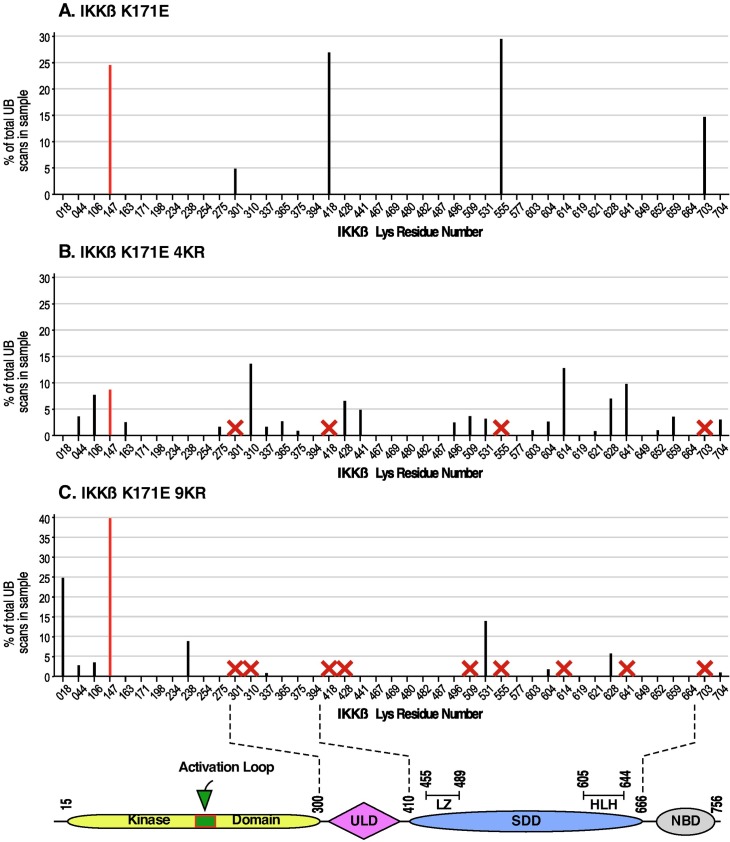
Identification of ubiquitination sites in IKKβ. As described in Materials and Methods, ubiquitination sites in IKKβ were detected by LC-MS/MS. All Lys residues in the IKKβ primary sequence are indicated on the x-axis. For each ubiquitination site detected, the magnitude of ubiquitination is indicated as a percentage of the total IKKβ ubiquitination. Sites representing < 2.5% of the total IKKβ ubiquitination are not shown. (A) IKKβ K171E. As described previously [5], K147 (shown in red) was identified as the primary site of K63-linked ubiquitination, and the sites K301, K418, K555 and K703 were also identified. (B) IKKβ K171E 4KR. The sites K301, K418, K555 and K703 identified in (A) were removed by mutagenesis, as shown by each red X. Many new sites of ubiquitination were now detected as shown. (C) IKKβ K171E 9KR. Five additional ubiquitination sites identified in (B) were again mutated, including K310R, K428R, K509R, K614R, and K641, as shown by each red X. One minor site identified in (B), K531, now became more prominent, and new sites at K018 and K238 now appeared. However, the ubiquitination site K147 now appears as the major site observed.

Fig 3 presents the results of LC-MS/MS analysis of cells expressing IKKβ proteins with the activating K171E mutation. In our initial study [5], major ubiquitination was identified at K147, K301, K418, K555, and K703 (Fig 3A). After determining that K147 is required for the kinase activation that is conferred by the K171E mutation, we analyzed additional samples to determine changes in ubiquitination after removing the other sites K301, K418, K555, and K703, as shown for IKKβ K171E 4KR (Fig 3B). Surprisingly, even with K301, K418, K555, and K703 removed (shown by “x”), many new sites of ubiquitination were observed. Of these sites, we chose to mutate additionally K310, K428, K509, K614 and K641, creating the protein IKKβ K171E 9KR, for which the summary of LC-MS/MS is presented (Fig 3C). As mentioned previously in the presentation of Fig 2C, Lanes 5 and 6, little overall reduction in total ubiquitination was observed. In the LC-LS/MS data for IKKβ K171E 9KR (Fig 3C), significant site preference was now observed for ubiquitination at K147, which now reached 40% of the total. The remainder of the ubiquitination was now distributed over sites previously not observed, or more minor in abundance. Recalling the story by Dr. Suess entitled “*The 500 Hats of Bartholomew Cubbins*,” in which Bartholomew is repeatedly ordered to remove his hat only to have another one mysteriously appear, no further effort was made to remove additional sites of ubiquitination beyond the 9KR series of constructs in IKKβ.

### Signaling pathways activated by IKKβ

**Fig 4:**
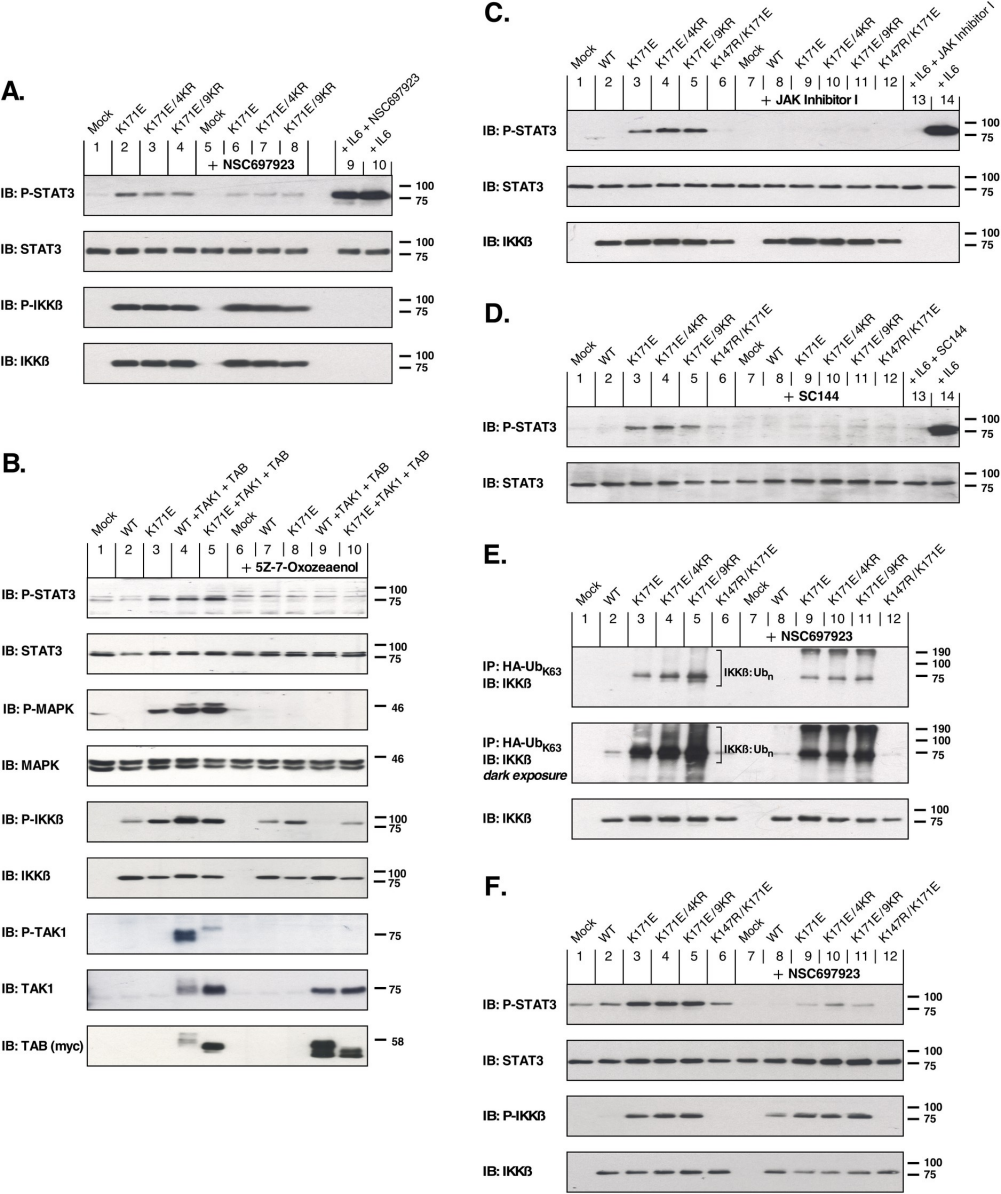
Examination of STAT3 activation by activated mutants of IKKβ. (A) Requirement for K63-linked ubiquitination. HEK293T cells expressing indicated IKKβ constructs were treated with 2μM NSC697923 for 2 h to inhibit UBE2N (Ubc13). STAT3 activation (1^st^ panel) was detected by immunoblotting for phospho-Tyr705-STAT3, with total STAT3 shown below. The 3^rd^ panel shows IKKβ kinase activation by immunoblotting for phospho-S177/S181 IKKβ, with total IKKβ shown below. The addition of exogenous IL-6 (10 ng/ml, 10 min) results in robust STAT3 activation (Lane 10) which is only marginally affected by treatment with the inhibitor NSC697923 (Lane 9). An empty lane is included between Lanes 8 and 9 due to the intensity of the signal in Lane 9. (B) Requirement for TAK1 activation. HEK293T cells expressing various combinations of IKKβ, TAK1 and TAB1 proteins were treated with 10 μM (5Z)-7-Oxozeaenol for 2 h to inhibit TAK1 activity. 1^st^ Panel: STAT3 activation is shown by immunoblotting for phospho-Tyr705-STAT3 and reveals that TAK1 activity is required for STAT3 activation, whether in response to IKKβ K171E (compare Lanes 3 and 8), or IKKβ WT activated by overexpression of TAK1 + TAB (compare Lanes 4 and 9). Total STAT3 is shown immediately below. 3^rd^ Panel: MAPK activation, shown by immunoblotting for phospho-T202/Y204-MAPK, is also dependent upon TAK1 activity similar to STAT3 activation. Total MAPK is shown immediately below. 5^th^ Panel: IKKβ kinase activation is shown by immunoblotting for phospho-S177/S181 IKKβ, with total IKKβ shown immediately below. Panels 7–9: Controls are presented for TAB and TAK1 expression and activation using phospho-T184/187-TAK1, total TAK1, and myc (9E10) to detect myc-tagged TAB. (C) Requirement for JAK activity. HEK293T cells expressing indicated IKKβ mutants were treated with the Janus kinase inhibitor JAK Inhibitor 1 (2 μM for 2 h). STAT3 activation is shown in the top panel by immunoblotting for phospho-Tyr705-STAT3 and reveals that JAK activity is required for STAT3 activation in response to activated IKKβ mutants (Lanes 3–5 compared with Lanes 9–11). The addition of exogenous IL-6 (10 ng/ml, 10 min) results in robust STAT3 activation (Lane 14) which is completely blocked by JAK Inhibitor 1 (Lane 13). (D) Requirement for GP130. A requirement for gp130 function, which serves as the β subunit of the IL-6-Receptor, was examined using the gp130 inhibitor SC144. HEK293T cells were starved and treated with 25 μM SC144 for ~20 h prior to a 2 h treatment with conditioned media from HEK293T cells expressing IKKβ mutants. STAT3 activation is shown in the top panel by immunoblotting for phospho-Tyr705-STAT3 and reveals that gp130 is required for STAT3 activation in response to activated IKKβ mutants (Lanes 3–5 compared with Lanes 9–11). The addition of exogenous IL-6 (10 ng/ml, 2 h) results in robust STAT3 activation (Lane 14) which is completely blocked by the gp130 inhibitor SC144 (Lane 13). Lysates from cells expressing the IKKβ mutants that generated the conditioned media were examined by immunoblotting to confirm IKKβ expression (data not shown). (E) Specific increase of K63-Ubiquitin-IKKβ by activated IKKβ mutants. HEK293T cells expressing indicated IKKβ mutants together with HA-tagged K63-only-Ubiquitin were immunoprecipitated with HA antiserum, and then immunblotted to detect total IKKβ. The higher MW bands of IKKβ suggest that activation by K171E, K171E 4KR, or K171E 9KR results in dramatically increased K63-conjugated Ub complexes, in comparison with IKKβ WT (compare Lanes 3–6 with Lane 2). This increase is largely, but not completely blocked, by the addition of 2μM NSC697923 for 2 h to inhibit UBE2N (Ubc13). (F) Requirement for K63-linked ubiquitination in IKKβ-deficient cells. IKKβ-deficient murine 3T3 cells expressing the indicated IKKβ mutants were treated with 5μM NSC697923 for 2 h to inhibit UBE2N (Ubc13)-catalyzed K63-linked ubiquitination. STAT3 activation (1^st^ panel) was detected by immunoblotting for phospho-Tyr705-STAT3, with total STAT3 shown below. The 3^rd^ panel shows IKKβ kinase activation by immunoblotting for phospho-S177/S181 IKKβ, with total IKKβ shown below.

The small-molecule inhibitor NSC697923 blocks the formation of K63-linked ubiquitin polymers that contribute to the activation of the IKK complex during inflammation-induced activation of NFκB [13–15]. The effect of NSC697923 on activation of STAT3 is shown in Fig 4A (1st panel), in which the K171E constructs–whether in the background of WT, 4KR, or 9KR–strongly activate phospho-STAT3 (lanes 2–4). In contrast, in the presence of NSC697923 the activation of phospho-STAT3 was reduced nearly to background levels (Lanes 6–8). Using an antiserum to detect phosphorylation within the activation loop of IKKβ (3^rd^ panel), the inhibition of K63-linked ubiquitination by NSC697923 showed no inhibitory effect. Thus, K63-linked ubiquitination is required for the downstream activation of STAT3 by IKKβ, but not for activation of IKKβ itself.

IKKβ is typically activated by the upstream protein kinase TAK1 [6]. (5Z)-7-Oxozeaenol, a selective and irreversible inhibitor, covalently binds to TAK1 and abolishes downstream TAK1-induced MAPK signaling [16]. Cells expressing IKKβ WT together with TAK1/TAB exhibited phosphorylated STAT3 similar to that resulting from overexpression of IKKβ K171E alone, or that resulting from IKKβ K171E together with TAK1/TAB (Fig 4B, 1st panel, Lanes 3–5). However, when treated with (5Z)-7-Oxozeaenol, STAT3 activation was impaired in these samples (Fig 4B, 1st panel, Lanes 8–10). Inhibition of TAK1 activity also abolished p44/42 MAPK (Erk1/2) signaling in cells expressing IKKβ K171E alone, or in cells expressing IKKβ constructs together with TAK1/TAB (Fig 4B, 3rd panel, compare Lanes 3–5 with 8–10). Notably, when coexpressed with TAK1/TAB, the phosphorylation of IKKβ WT was inhibited by the TAK1 inhibitor (Fig 4B, 5^th^ panel, Lane 4 vs. 9), whereas phosphorylation of IKKβ K171E was not (Fig 4B, 5^th^ panel, Lane 5 vs 10). These results demonstrate a requirement for TAK1 in the downstream activation of STAT3 in response to activated IKKβ.

### Signaling by IKKβ K171E requires JAKs and the IL-6 signal transducer gp130

STAT3 is typically activated via IL-6-induced dimerization of the gp130 subunits of IL-6R, subsequently activating the JAKs to phosphorylate STAT3 [9]. In order to examine the requirement for JAK kinases, the K171E mutants–in the background of WT, 4KR, or 9KR–were examined in the absence or presence of JAK Inhibitor I, which is an early inhibitor of JAK family members based on a benzimidazole core. Fig 4C (1^st^ panel) shows the resulting inhibition of STAT3 phosphorylation in the presence of JAK Inhibitor 1 (Lanes 3–5 vs Lanes 9–11). In this experiment, we also showed that JAK Inhibitor 1 inhibited phosphorylation of STAT3 in response to exogenous IL-6 (Lane 13 vs 14).

We also considered whether an autocrine signaling response to IL-6 was induced by IKKβ K171E mutants, a model which will be examined further below. Fig 4D examines this possibility, as well as the requirement for the IL-6 signal transducer gp130. Cells were starved and treated with the gp130 inhibitor SC144, after which they were incubated with conditioned media from HEK293T cells expressing the IKKβ K171E mutants. As seen (1^st^ panel), conditioned media from cells expressing the K171E mutants–in the background of WT, 4KR, or 9KR–clearly stimulated phospho-STAT3, and this was completely blocked by the presence of SC144 (Lanes 3–5 vs Lanes 9–11). Again, as a control, SC144 completely inhibited the response to exogenously added IL-6 (Lane 13 vs 14).

The data presented in Fig 4, Panels C and D, clearly demonstrate that the downstream effects of IKKβ K171E mutants require the activity of JAK proteins acting in concert with the IL-6 signal transducer gp130.

### Inhibition of K63-linked ubiquitination

To further examine the ubiquitination of the IKKβ K171E mutants, we exploited an HA-tagged ubiquitin clone in which all Lys residues except for K63 were removed by mutagenesis. When coexpressed with IKKβ K171E proteins, this should result in a highly enriched signal for K63-linked ubiquitination. The results of this experiment are shown in Fig 4E (1^st^ and 2^nd^ panels), in the absence or presence of NSC697923 to inhibit the E2 ubiquitin conjugating enzyme UBE2N (Lanes 3–5 vs Lanes 9–11). Although NSC697923 largely inhibits ubiquitination of IKKβ in this assay, it does not eliminate it completely. In the darker exposure, there is a very strong signal of polyubiquitinated IKKβ, indicated by the brackets, which is attenuated in the presence of the UBE2N inhibitor. These results demonstrate that the apparent ubiquitination of IKKβ K171E mutants occurs significantly by a K63-linked pathway, although not exclusively. Of note is the massive ubiquitination of the IKKβ K171E mutants in comparison with IKKβ WT, which is almost devoid of a ubiquitination signal except in the dark exposure (Fig 4E, 2^nd^ panel, Lane 2).

### Requirement for K63-linked ubiquitination for IKKβ-dependent STAT3 activation in IKKβ-deficient cells

To rule out the possibility that the presence of endogenous IKKβ might have a major effect in the preceding experiments, we also utilized IKKβ-deficient murine cells expressing IKKβ mutants. These were examined in the absence or presence of the small-molecule inhibitor NSC697923 to inhibit K63-linked ubiquitination. The effect of NSC697923 on activation of STAT3 is shown in Fig 4F (1st panel), in which the K171E constructs–whether in the background of WT, 4KR, or 9KR–activate phospho-STAT3 (lanes 3–5). In contrast, in the presence of NSC697923 the activation of phospho-STAT3 was reduced nearly to background levels (Lanes 9–11). Using an antiserum to detect phosphorylation within the activation loop of IKKβ (3^rd^ panel), the inhibition of K63-linked ubiquitination by NSC697923 showed little inhibitory effect. Thus, in cells that are IKKβ-deficient, K63-linked ubiquitination is required for the downstream activation of STAT3 by IKKβ, but not for activation of IKKβ itself.

### Proliferation of IL-6-dependent INA-6 cells by IKKβ K171E mutants

The K171E mutation of IKKβ was initially identified in multiple myeloma [2] and spleen marginal zone lymphoma [3], and the K171R mutation in mantle cell lymphoma [4]. In order to investigate the biological significance of the K171E mutation, we exploited the murine myeloid 32D cell line which depends on exogenous IL-3 for proliferation and viability [17], and the INA-6 cell line, which is dependent upon exogenous IL-6 for proliferation [18]. In experiments with 32D cells, 32D cells stably expressing IKKβ WT, K171E and K171E 4KR, we were unable to demonstrate significant proliferative responses (data not shown).

**Fig 5:**
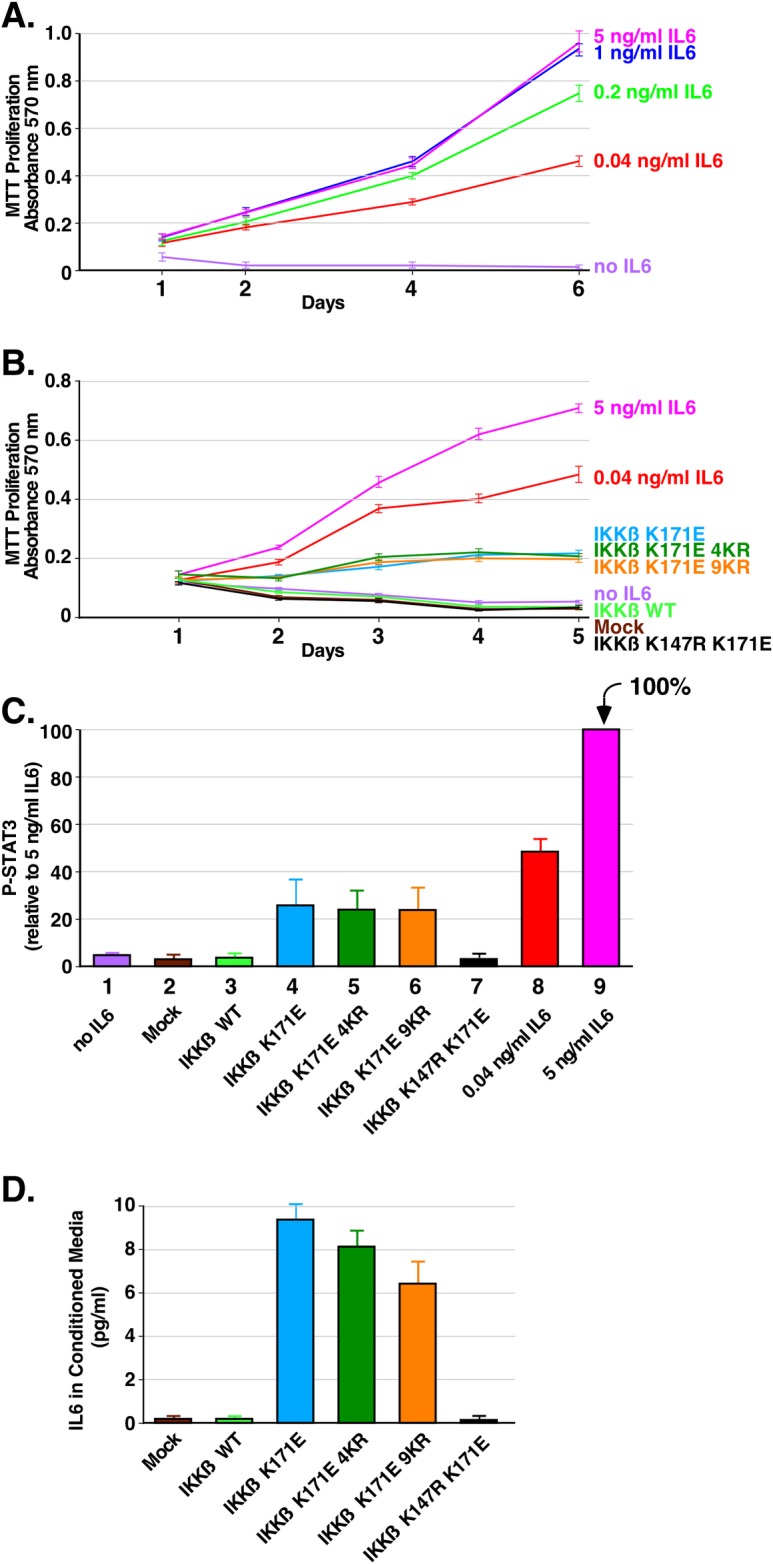
Assessing the oncogenic potential of the K171E mutation in IKKβ on the IL-6 -dependent INA-6 cell line. The human myeloma cell line INA-6 is completely dependent on exogenous IL-6 for growth and proliferation. (A) IL-6 concentration dependence of the INA-6 cells. Triplicate cultures of cells were grown in RPMI 1640 with 10% FBS and various concentrations of IL-6 (5, 1, 0.2, 0.04 and 0 ng/ml). Duplicate samples were collected at 1, 2, 4 and 6 days and assayed by MTT metabolic assay indicating the number of viable cells. Error bars show the standard deviation (B) Proliferation of INA-6 cells treated with conditioned media. Triplicate cultures of INA-6 cells were incubated in media collected from HEK293T cells expressing IKKβ derivatives. 10% FBS was added to the starvation media. Duplicate samples were collected at 1, 2, 3, 4 and 5 days and assayed by MTT metabolic assay. Control samples were treated 5, 0.04 and 0 ng/ml of IL-6 as indicated. Error bars show the standard deviation (C) STAT3 activation induced by conditioned media from cells expressing IKKβ mutants. INA-6 cells were incubated as in B. for 48 h. Cells were lysed and immunoblotted for P-STAT3. Triplicate immunoblots were quantitated with 5 ng/ml of IL-6 set at 100%. Error bars show the standard deviation. (D). Detection of IL-6 in conditioned media from cells expressing IKKβ mutants. Using a sensitive ELISA kit for IL-6, the conditioned media was assayed in triplicate from each of two independent experiments. Concentrations were determined from a standard curve of IL-6.

Experiments with INA-6 cells, however, were much more revealing as presented in Fig 5. First, we established a dose response curve to IL-6 (Panel A), showing a very sensitive proliferative response by INA-6 cells. Next, we collected conditioned media from HEK293T cells expressing IKKβ mutants which, as shown above (Fig 4, Panel D), were capable of stimulating phospho-STAT3 when applied to HEK293T cells. When the conditioned media samples were applied to INA-6 cells in the absence of IL-6, proliferation was observed in response to IKKβ K171E, K171E 4KR, and K171E 9KR, which were essentially indistinguishable from one another. In contrast, neither IKKβ WT, nor kinase-dead IKKβ K147R K171E, were able to stimulate a proliferative response in INA-6 cells above the background condition of no IL-6 (Fig 5B). Phospho-STAT3 stimulation was also examined in the INA-6 cells treated with conditioned media for 48 h (Fig 5C). These results suggested that we might be able to measure IL-6 directly by ELISA in the conditioned media of the cells (Fig 5D). This showed low but significant amounts of IL-6 in the conditioned media from cells expressing the activated proteins IKKβ K171E, K171E 4KR, and K171E 9KR, which exhibited 9.5, 8.2, and 6.5 pg/ml of IL-6, respectively. As expected, no IL-6 was detectable in the conditioned media from mock cells, cells expressing IKKβ WT, or cells expressing kinase-dead IKKβ K147R K171E.

Overall, the data in Fig 5 unequivocally demonstrate the secretion of either IL-6 itself, or another related cytokine, capable of eliciting a proliferative response in the IL-6 dependent cell line INA-6, in response to the activated IKKβ K171E proteins, with similar response observed for IKKβ K171E, IKKβ K171E 4KR, and IKKβ K171E 9KR.

### Molecular functions activated by IKKβ K171E mutants

In order to probe changes in the abundancy of total proteins and of ubiquitin-conjugated proteins as a consequence of activated IKKβ K171E mutants, we employed a proteomics approach. Large samples of 1.8x10e7 HEK293T cells were prepared expressing IKKβ WT or IKKβ K171E 9KR, in the presence of the HA-tagged K63-only-Ub. Four replicates of each sample were prepared. These lysates were immunoprecipitated with anti-HA mAB, subjected without further purification or enrichment by LC-MS/MS, and spectral data was then analyzed by MaxQuant label free quantitation (LFQ) [19]. This approach depended upon the hypothesis that the population of detectable proteins in these two groups would be different, as measured by the amounts and identities of the proteins. We wished to determine what pathways and functions are enriched in each group.

**Fig 6:**
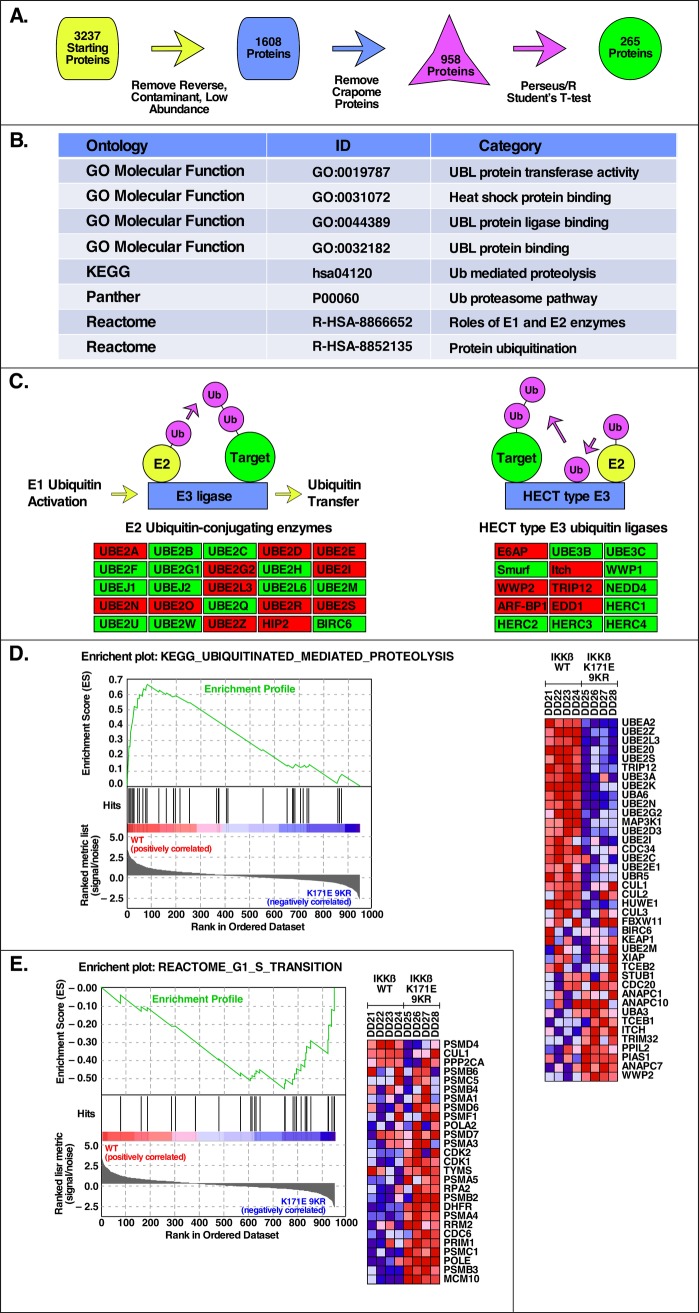
Network analysis in response to activated mutants of IKKβ. (A) Proteomic Analysis Pipeline. Schematic representation of the analysis steps for proteomic mass spectrometry data that uses MaxQuant Label Free Quantification values of detected proteins as input and generates a list of significantly differentially abundant proteins (Benjamini-Hochberg adjusted p-value < 0.05) as output. (B) Top Biological Categories in Proteomic Analysis. Gene Ontology, KEGG, Panther, and Reactome pathways/categories identified as significantly enriched by WebGestalt (FDR < 0.05) for the list of differentially abundant proteins identified in (A). (C) Pathway Level Representation of Select Differentially Abundant Proteins. Representative sub-processes of the significantly enriched Ubiquitin Mediated Proteolysis KEGG category from (B) that have a high percentage overlap with the differentially abundant protein list. Red boxes indicate proteins with significantly different abundance between the *IKKβ* WT and *IKKβ* K171E 9KR sample groups. (D) Top GSEA Result for *IKKβ* WT. Using the list of 958 proteins identified in the two groups as input, the GSEA identified Gene set (KEGG Ubiquitin Mediated Proteolysis) with the highest Enrichment Score (ES) in *IKKβ* WT along with a heatmap of the top genes contributing to the ES. (E) Top GSEA Result for *IKKβ* K171E 9KR. As in (D), the Gene set (Reactome G1 to S Transition) with the highest ES in *IKKβ* K171E 9KR along with the corresponding heatmap. In the heatmaps, the range of colors (red, pink, light blue, dark blue) shows the range of expression values for each gene in each sample (high, moderate, low, lowest).

As presented in Fig 6A, MaxQuant LFQ protein data [19] containing 3,237 initial proteins were analyzed using the Perseus Software/R environment [20, 21] and, after removal of low abundancy and Crapome proteins [22], 958 proteins remained. Using a Student’s T-test and false discovery threshold of 0.1, 265 proteins were identified as significantly differentially abundant between the IKKβ WT and IKKβ K171E 9KR groups (see Supplemental Information). The major Gene Ontology, KEGG, and Panther categories identified after performing an enrichment analysis on these protein groups are presented in Fig 6B and are primarily related to ubiquitin ligases, ubiquitin-like protein transferases and binding, and ubiquitin proteasomal degradation pathways. Analysis also revealed significant association with several E2 components, including UBE2N but also several others, as well as several HECT type E3 ligases. A partial summary of these data is presented in Fig 6C. Analysis by GSEA, or Gene Set Enrichment Analysis [23, 24], yielded several particularly interesting gene sets, two of which are presented here: Fig 6D presents proteins identified in KEGG UBIQUITINATED MEDIATED PROTELYSIS that were positively correlated with IKKβ WT samples (i.e. increased abundance relative to IKKβ K171E 9KR); and Fig 6E presents proteins identified in REACTOME G1 S TRANSITION that were negatively correlated with IKKβ WT samples (i.e. decreased abundance relative to IKKβ K171E 9KR). IKKβ K171E 9KR sample. These proteins, indicating a role in progression through G1/S of the cell cycle, are consistent with the overall proliferative responses described previously for activating IKKβ K171E mutants.

## Discussion

We present experiments probing signaling by the oncogenic K171E mutation of IKKβ, originally identified in hematological malignancies [2–4]. The K63-linked ubiquitination of K147 is upregulated by the K171E mutation, which induces activation of STAT3. Throughout, we have focused our efforts on detecting Tyr705 phosphorylation of STAT3 in response to K171E mutations in IKKβ, since cytokine-mediated activation of phosphorylation at this site has been extensively shown to lead to nuclear translocation of STAT3.

Initially, we had hoped we might create an IKKβ K171E mutant in which the only remaining site of ubiquitination would be K147. In this quest, we first eliminated K301, K418, K555, and K703, sites initially identified as ubiquitination sites, to create the 4KR mutants. Although this resulted in a slight relative increase in utilization of K147 for ubiquitination, many additional sites of ubiquitination were now observed. We then eliminated K310, K428, K509, K614, and K641 which, when combined with the 4KR mutants, created the 9KR mutants. Although K147 was now preferentially utilized, we still saw the appearance of many additional sites of ubiquitination. We found few changes in the ability of these different mutants to activate downstream pathways; as discussed below, IKKβ K171E, IKKβ K171E 4KR, and IKKβ K171E 9KR all exhibited relatively similar activation of downstream pathways.

### K63-linked ubiquitination in IKKβ, TAK1 and BRAF

K63-linked ubiquitination stabilizes signaling functions of proteins and is an indispensable modification for the activation of innate and acquired immune responses, including DNA damage repair pathways [25]. Activation of the IKK complex and canonical NFκB signaling depends upon K63-linked ubiquitination of TAK1 and the linear ubiquitination of NEMO [26, 27]. We show that the activating mutation K171E of IKKβ upregulates K63-linked ubiquitination at K147, an evolutionarily conserved residue that modulates the kinase function of IKKβ [5]. K63-linked ubiquitination has also been reported in other noteworthy kinases at sites homologous to the K147 site in IKKβ. One example is K63-linked ubiquitination at K158 in TAK1, required for NFκB signaling during inflammatory responses [28–30]. Another example is K63-linked ubiquitination at K578 in BRAF V600E [31], which serves as an oncogenic driver in melanoma, lung and colorectal cancer [32]. Therefore, while the outcome of K63-linked ubiquitination at these sites may differ from that described here for IKKβ, it is significant that multiple kinases undergo regulatory K63-linked ubiquitination at this homologous residue to modulate inflammation and oncogenicity.

### Signaling pathways activated by IKKβ K171E

**Fig 7:**
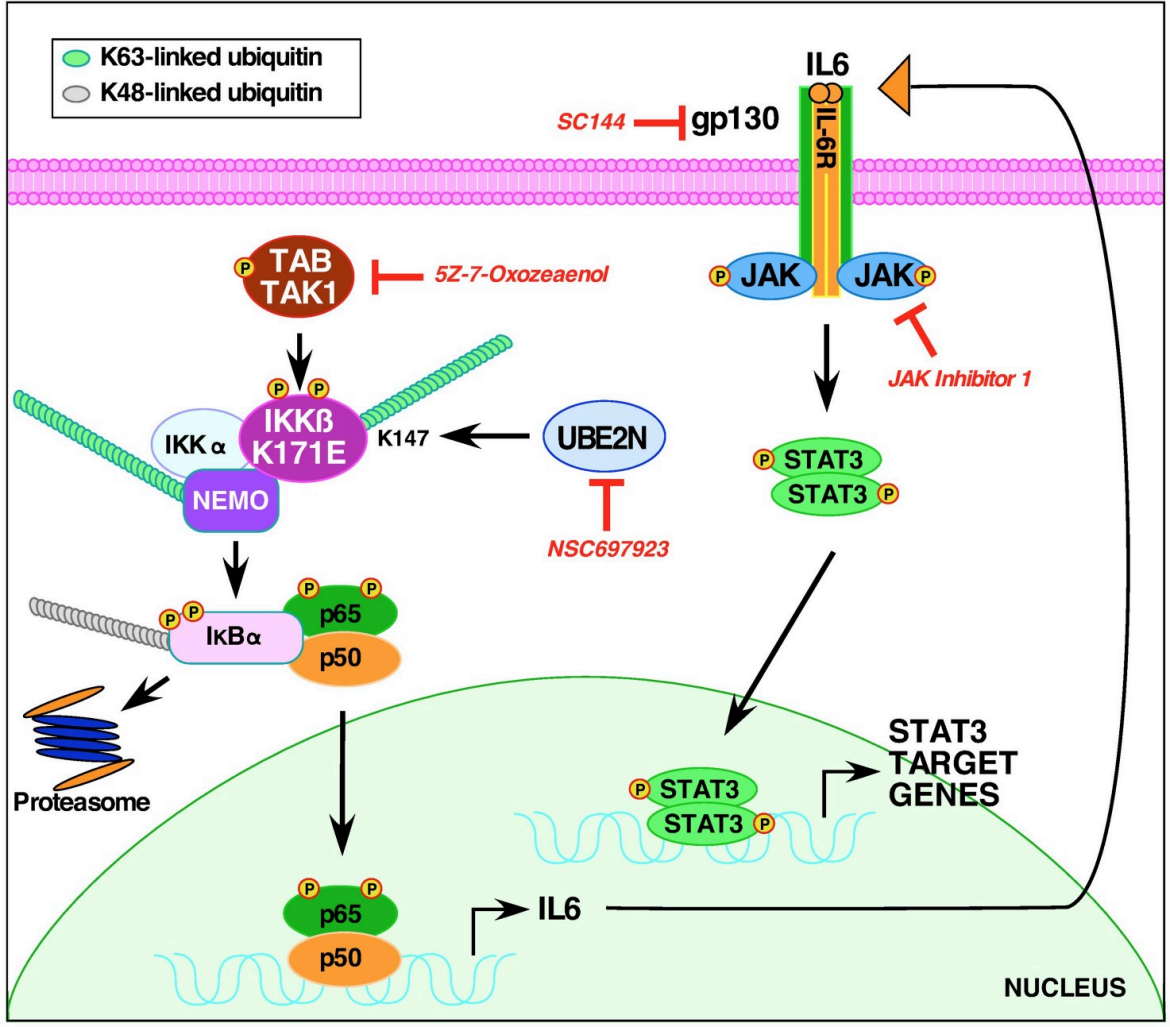
Signaling pathways activated by K171E IKKβ. A model is presented for signaling by the oncogenic mutation K171E of IKKβ, identified in hematological malignancies, which integrates the data presented here. Normally, inflammatory cytokines activate NFκB and signal the K63-linked ubiquitination of TAK1 and NEMO, leading to the activation of the IKK complex and NFκB nuclear translocation. The K171E mutation of IKKβ instead leads to the K63-linked ubiquitination of K147 by UBE2N (Ubc13), as shown by the inhibitor NSC697923, and this activation is dependent upon the activity of TAK1, as shown by the inhibitor 5Z-7-Oxozeaenol. The IKKβ K171E mutants establish an autocrine loop dependent upon the secretion of IL-6, binding to the IL-6 receptor, as shown by the inhibitor SC144 which inhibits the β-subunit gp130. The involvement of the JAK kinase family (JAK1, JAK2, JAK3, TYK2) in this system is shown by the inhibitor JAK Inhibitor 1, which also inhibits the appearance of phospho-STAT3 in response to IKKβ K171E mutants.

A model integrating our results in presented in Fig 7. First, the K63-linked ubiquitination of IKKβ is required for activation of STAT3, as shown by the use of the inhibitor NSC697923 [15]. This result is consistent with our earlier work [5]. Surprisingly, although TAK1 activity was not required for activation of K171E kinase activity, as indicated by S177/S181 phosphorylation of the IKKβ activation loop, TAK1 activity was required to observe the subsequent activation of STAT3. This was revealed through the use of the TAK1 inhibitor (5Z)-7-Oxozeaenol [16, 33]. Clearly, the IKKβ K171E mutants activate an autocrine loop in which IL-6 is secreted and subsequently binds to the IL-6 receptor complex gp130, resulting in JAK activation. This was shown using the gp130 inhibitor SC144 [34, 35] and using the JAK/TYK2 inhibitor JAK Inhibitor 1 [36–38], both of which inhibited the appearance of phospho-STAT3 in response to IKKβ K171E mutants. The release of either IL-6 or a closely related molecule was directly demonstrated by collecting conditioned media from HEK293T cells expressing the IKKβ K171E mutants and showing: 1) these conditioned media resulted in STAT3 activation when applied to naive cells; and 2) these conditioned media stimulated proliferation and STAT3 activation of the IL-6-dependent cell line INA-6 [18] in the absence of exogenous IL-6.

We also employed a proteomics approach to compare cells expressing IKKβ WT in comparison with IKKβ K171E 9KR, examining all proteins recovered that were labeled with a coexpressed HA-tagged K63-only-Ub. These proteins were analyzed without further purification or enrichment by LC-MS/MS and MaxQuant label free quantitation [19]. We found that the populations of detectable proteins in these two groups were different as measured by the amounts and identities of the proteins. The major Gene Ontology, KEGG, and Panther categories identified were primarily related to ubiquitin ligases, ubiquitin-like protein transferases and binding, and ubiquitin proteasomal degradation pathways. Significant association was revealed with several E2 components, including UBE2N but also several others, as well as several HECT type E3 ligases. Among a number of different gene sets identified, proteins involved in ubiquitinated mediated proteolysis had abundance increased in the IKKβ WT sample group, whereas proteins associated with cell cycle progression had abundance increased in the IKKβ K171E 9KR sample group. These analyses reveal a major disruption of cellular ubiquitination functions by the activating mutation K171E, and reinforce the other experiments presented here that demonstrate an IL-6-independent proliferative advantage conferred by IKKβ K171E. This is also consistent with the activation of phospho-STAT3, shown throughout this work, which plays a major role as a transcriptional activator for many genes including those involved in the regulation of cell proliferation [39].

### Potential clinical applications

The identification of IKKβ mutations at position 171 in hematological malignancies [2–4] suggests that their oncogenic potential depends on a specific cellular phenotype and genetic program. Patients with multiple myeloma, for instance, are usually treated with Bortezomib, a proteasome inhibitor that attenuates aberrant NFκB signaling. However, Bortezomib resistance is inevitable [40], demonstrating that Bortezomib-mediated inhibition of NFκB signaling may not be sufficient to achieve successful treatment. Cancers harboring K171-mutated IKKβ are likely to also exhibit activated STAT3 and p44/42 MAPK (Erk1/2), suggesting the possibility of using MAPK (Erk1/2) and JAK inhibitors, or specific ubiquitination inhibitors.

## Materials and methods

### Plasmid constructs

Starting with expression plasmids for IKKβ WT and IKKβ K171E [5], the derivatives IKKβ WT 4KR (K301R, K418R, K555R and K703R) and IKKβ K171E 4KR were generated by QuikChange site-directed mutagenesis (Agilent). These were utilized for further mutagenesis to create the IKKβ WT 9KR (K301R, K310R, K418R, K428R, K509R, K555R, K614R, K641R, K703R) and IKKβ K171E 9KR plasmids (Fig 1). Kinase-dead derivatives were made by introduction of the K147R mutation. hTAK1 in pCMV6-XL5 was a gift from Dr. Leslie Thompson (UC Irvine) and myc-TAB1 in pcDNA3 was a gift from Dr. Carol Prives (Columbia University).

The HA-Ub3 and HA-Nemo plasmids were described previously [5]. The HA-K63-only-Ub (K6R, K11R, K27R, K29R, K33R, K48R) plasmid was also generated by QuikChange site-directed mutagenisis (Agilent) in a pcDNA3 background; two rounds of mutagenesis were conducted, introducing the additional mutations together with new silent restriction sites, AgeI and NruI. The initial HA-Ub-K48R plasmid [41] was a gift from Cam Patterson (University of Arkansas), obtained from Andrea Carrano (UC SanDiego).

### Cell culture and INA-6 viability assay

HEK293T were grown in DMEM with 10% FBS and maintained in 10% CO_2_ at 37°C. Cells were transfected with plasmid DNA using calcium phosphate precipitation at 3% CO_2_. Approximately 18 h after transfection, cells were starved overnight before collecting in RIPA Lysis Buffer, as previously described [10]. The IL-6-dependent multiple myeloma INA-6 cell line [18] was a generous gift from Dr. Erming Tian (University of Arkansas) and grown in RPMI 1640 with 10% FBS, 10% Marrow Max Bone Medium (Gibco Life Technologies), 10 ng/ml hIL-6 (R&D Systems) and maintained in 5% CO_2_ at 37°C. IKKβ knockout murine 3T3 cells [42] were kindly provided by Dr. Alexander Hoffmann (UCLA) and maintained in DMEM with 10% CS with 10% CO_2_.

To determine the IL-6 concentration dependence, INA-6 cells were seeded at an initial density of 2.5 x 10^5^ cells/ml in media lacking Marrow Max Bone Medium, containing the indicated concentrations of hIL-6. Samples of triplicate cultures were removed and examined by MTT metabolic assay at each time point.

For MTT metabolic assays, 500 μl of cultures were transferred in duplicate to a 24-well TC plate and incubated with 50 μl of 5 mg/mL of thiazolyl blue tetrazolium bromide (MTT) (Sigma) at 37°C in 5% CO_2_ for 4 h, after which 500 μl of 0.04 M HCl in isopropanol was added and incubated again for at least 30 min. Absorbance was measured at 570 nm.

IKKβ induced proliferation of INA-6 cells was determined by treating the cells with starvation media (RPMI1640) from HEK293T cells expressing IKKβ mutants. INA-6 cells were seeded at 3 x 10^4^ cells/ml. FBS was added to sterile filtered starvation media prior to treatment at a 10% final concentration. Samples of triplicate cultures were removed and examined by MTT metabolic assay at each time point.

To determine STAT3 activation, INA-6 cells seeded at 1.4 x 10^5^ cells/ml were lysed in RIPA after 48 h incubation with media from HEK293T cells expressing IKKβ plasmids, prepared as described above. Lysates were immunoblotted as described below. Triplicate immunoblots were quantitated.

### Electrophoresis, immunoblotting and additional reagents

Lysates were collected in RIPA lysis buffer containing inhibitors 10 ng/ml Aprotinin, 1 mM PMSF, 1 mM Na3VO4, 20 mM β-glycerol-phosphate, and 5 mM N-ethyl-maleimide, and proteins were separated by 10% or 12.5% SDS-PAGE followed by transfer to Immobilon-P membrane. For immunoprecipitations, 400 μg of total protein was incubated with αHA (F7) overnight and collected with Protein A Sepharose (Sigma) or Pierce Protein A/B Magnetic Beads (Thermo Fisher Scientific). Immunoblotting reagents were from the following sources: antibodies against IKKβ (H-4), IKKβ (G-8), HA-probe (F-7), STAT3 (C-20), Myc (9E10), and TAK1 (M-579) from Santa Cruz Biotechnology; Phospho-IKKα/β (Ser176/180) (16A6), Phospho-STAT3 (Tyr705) (D3A7), and Phospho-TAK1 (T184/187) (4531S) from Cell Signaling Technology; HRP anti-mouse, HRP anti-rabbit, and Enhanced Chemiluminescence (ECL) reagents from GE Healthcare. Other reagents included: NSC697923 from Santa Cruz Biotechnology; JAK Inhibitor 1 from Calbiochem; MG132 and (5Z)-7-Oxozeaenol from Tocris Bioscience; SC144 from Sigma-Aldrich; recombinant human IL-6 from R&D Systems. IL-6 concentrations in conditioned media were determined by ELISA, using Human IL-6 Quantikine ELISA D6050 from R&D Systems according to the manufacturer’s protocol.

### Mass spectrometry and network analysis

For identification of ubiquitination sites, HEK293T cells were transfected with the indicated IKKβ plasmids together with HA-Ub_3_ and HA-NEMO plasmids. Cells were starved overnight and treated with 10 μM MG132 for at least 48 h before collection. Lysates were collected in RIPA and analyzed as previously described [5]. For IKKβ K171E, data presented in Fig 3A were included previously in Table 1 of [5]. For IKKβ K171E 4KR (Fig 3B), and IKKβ K171E 9KR (Fig 3C), lysates were prepared in Sonication Buffer and subjected to sonication as described [43]. Lysates were immunoprecipitated with anti-IKKβ (H-4), and collected using Pierce Protein A/G Magnetic Beads prior to analysis by LC-MS/MS.

For the comparative analysis of IKKβ WT and IKKβ K171E 9KR, quadruplicate sets of 1.8x107 HEK293T cells were transfected, together with HA-K63-only-Ub. Lysates were sonicated, immunoprecipitated with anti-HA (F-7), and collected using Pierce Protein A/G Magnetic Beads. Dried samples were reconstituted with 2% acetonitrile, 0.1% formic acid and analyzed by LC-MS/MS using a Proxeon EASY nanoLC system (Thermo Fisher Scientific) coupled to a Q-Exactive Plus mass spectrometer (Thermo Fisher Scientific). Peptides were separated using an analytical C_18_ Acclaim PepMap column 0.075 x 500 mm, 2 μm particles (Thermo Fisher Scientific) in a 94-min linear gradient of 2–28% solvent B at a flow rate of 300 nL/min. The mass spectrometer was operated in positive data-dependent acquisition mode. MS1 spectra were measured with a resolution of 70,000, an AGC target of 1e6 and a mass range from 350 to 1700 m/z. Up to 12 MS2 spectra per duty cycle were triggered, fragmented by HCD, and acquired with a resolution of 17,500 and an AGC target of 5e4, an isolation window of 1.6 m/z and a normalized collision energy of 25. Dynamic exclusion was enabled with duration of 25 sec.

LC-MS/MS spectra were analyzed using the quantitative proteomics software package MaxQuant which was used for peak detection, scoring of peptides, protein identification and label free quantification [19, 20]. The MaxQuant output of quantified values for each protein in each sample was used as input to the Perseus software package which was used to remove proteins identified as contaminants or from a database of reverse proteins, impute absent values, log transform, and perform a width adjustment normalization on the data [21]. An additional filtering step removed proteins identified as bound non-specifically during the affinity purification. This list was generated by a search of the Crapome database with the parameters: HEK293 (cell type), HA (affinity tag), and magnetic beads (affinity support) [22].

To identify proteins with significantly different abundance between the two sample groups, a Student’s T-test, within the Perseus software, was performed on all proteins. An adjustment of the p-values for multiple testing was made using the Benjamini-Hochberg method. To identify significantly enriched pathways and functions (FDR < 0.05), the list of differentially abundant proteins was used as input into the WebGestalt package within the R statistical computing environment [44, 45]. Gene Set Enrichment Analysis was performed on the complete list of identified proteins using the default parameters [23].

## Supporting information

S1 Table LFQ proteomic data. List of differentially abundant proteins and Label Free Quantification (LFQ) values, raw and processed, for all proteins identified in mass spectrometry experiments. Differentially abundant protein list contains the results of statistical tests (with multiple testing correction) comparing abundance between WT and K171E 9KR IKKβ sample groups. Processing of LFQ data includes data imputation, width adjustment normalization, and log2 transformation.
(XLSX)

S2 Table Pathway enrichment analysis. Results of functional and pathway enrichment analysis (Gene Ontology–Molecular Function, KEGG, Panther, Reactome, and GSEA) performed on significantly differentially abundant proteins between WT and K171E 9KR IKKβ sample groups.
(XLSX)

S3 Table Ubiquitinated peptides. List of proteins and quantified totals of ubiquitinated peptides in WT and K171E 9KR IKKβ sample groups sample identified in mass spectrometry experiments.
(PPTX)

S4 Table Input data for proteomic analysis. Raw data for all identified proteins in all WT and K171E 9KR IKKβ samples including annotation, identified peptide totals, and label free quantification values.
(XLSX)
