# Supplementary material for: Oncogenic mutations in IKKβ function through global changes induced by K63-linked ubiquitination and result in autocrine stimulation
Source: PLoS One. 2018 Oct 18;13(10):e0206014. doi: 10.1371/journal.pone.0206014 (PMC6193727; doi:10.1371/journal.pone.0206014)
Supplement: S3 Table — List of proteins and quantified totals of ubiquitinated peptides in WT and K171E 9KR IKKβ sample groups sample identified in mass spectrometry experiments. (PPTX) [file pone.0206014.s003.pptx]

## Slide 1
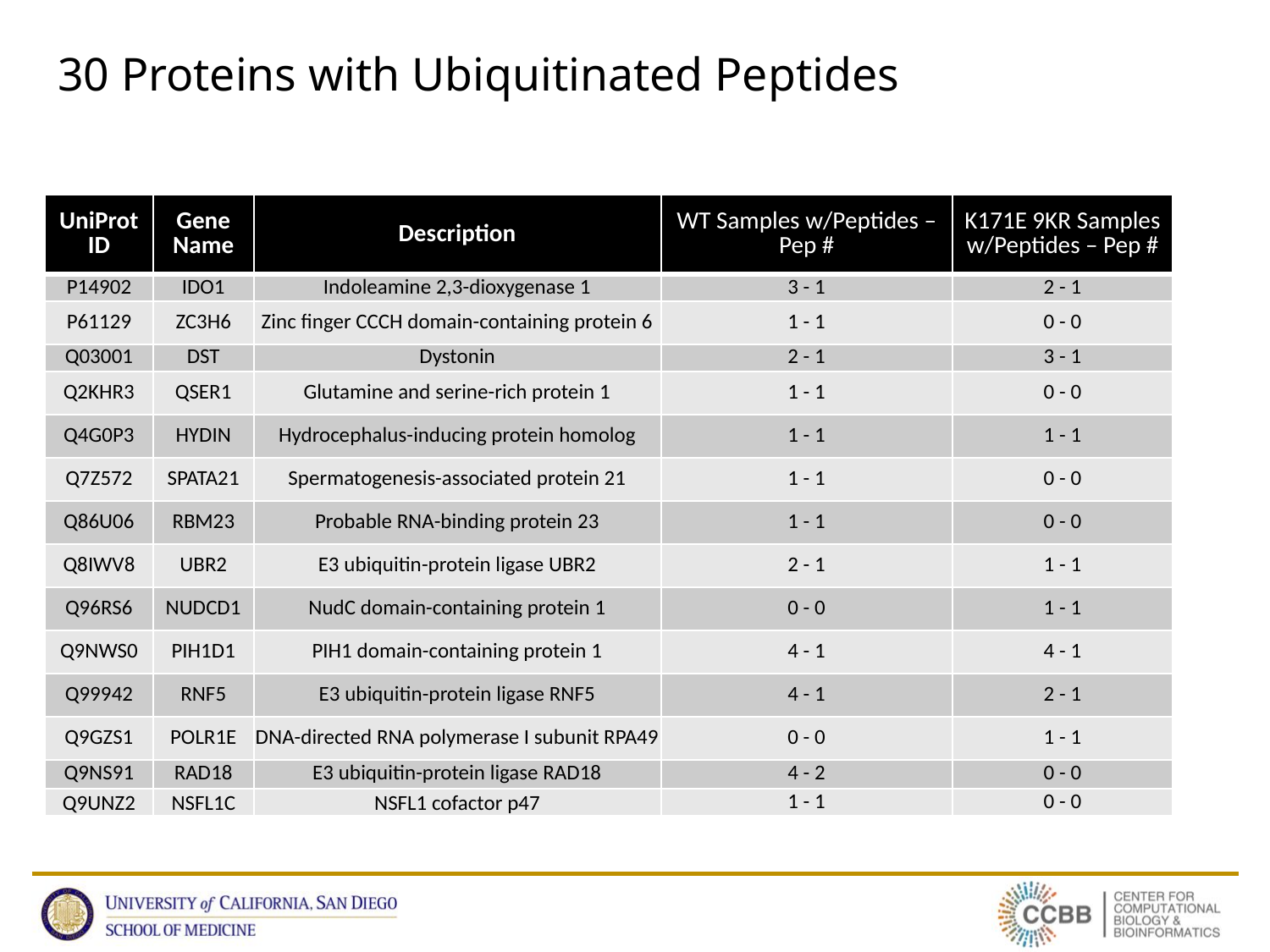

# 30 Proteins with Ubiquitinated Peptides
| UniProt ID | Gene Name | Description | WT Samples w/Peptides – Pep # | K171E 9KR Samples w/Peptides – Pep # |
| --- | --- | --- | --- | --- |
| P14902 | IDO1 | Indoleamine 2,3-dioxygenase 1 | 3 - 1 | 2 - 1 |
| P61129 | ZC3H6 | Zinc finger CCCH domain-containing protein 6 | 1 - 1 | 0 - 0 |
| Q03001 | DST | Dystonin | 2 - 1 | 3 - 1 |
| Q2KHR3 | QSER1 | Glutamine and serine-rich protein 1 | 1 - 1 | 0 - 0 |
| Q4G0P3 | HYDIN | Hydrocephalus-inducing protein homolog | 1 - 1 | 1 - 1 |
| Q7Z572 | SPATA21 | Spermatogenesis-associated protein 21 | 1 - 1 | 0 - 0 |
| Q86U06 | RBM23 | Probable RNA-binding protein 23 | 1 - 1 | 0 - 0 |
| Q8IWV8 | UBR2 | E3 ubiquitin-protein ligase UBR2 | 2 - 1 | 1 - 1 |
| Q96RS6 | NUDCD1 | NudC domain-containing protein 1 | 0 - 0 | 1 - 1 |
| Q9NWS0 | PIH1D1 | PIH1 domain-containing protein 1 | 4 - 1 | 4 - 1 |
| Q99942 | RNF5 | E3 ubiquitin-protein ligase RNF5 | 4 - 1 | 2 - 1 |
| Q9GZS1 | POLR1E | DNA-directed RNA polymerase I subunit RPA49 | 0 - 0 | 1 - 1 |
| Q9NS91 | RAD18 | E3 ubiquitin-protein ligase RAD18 | 4 - 2 | 0 - 0 |
| Q9UNZ2 | NSFL1C | NSFL1 cofactor p47 | 1 - 1 | 0 - 0 |

## Slide 2
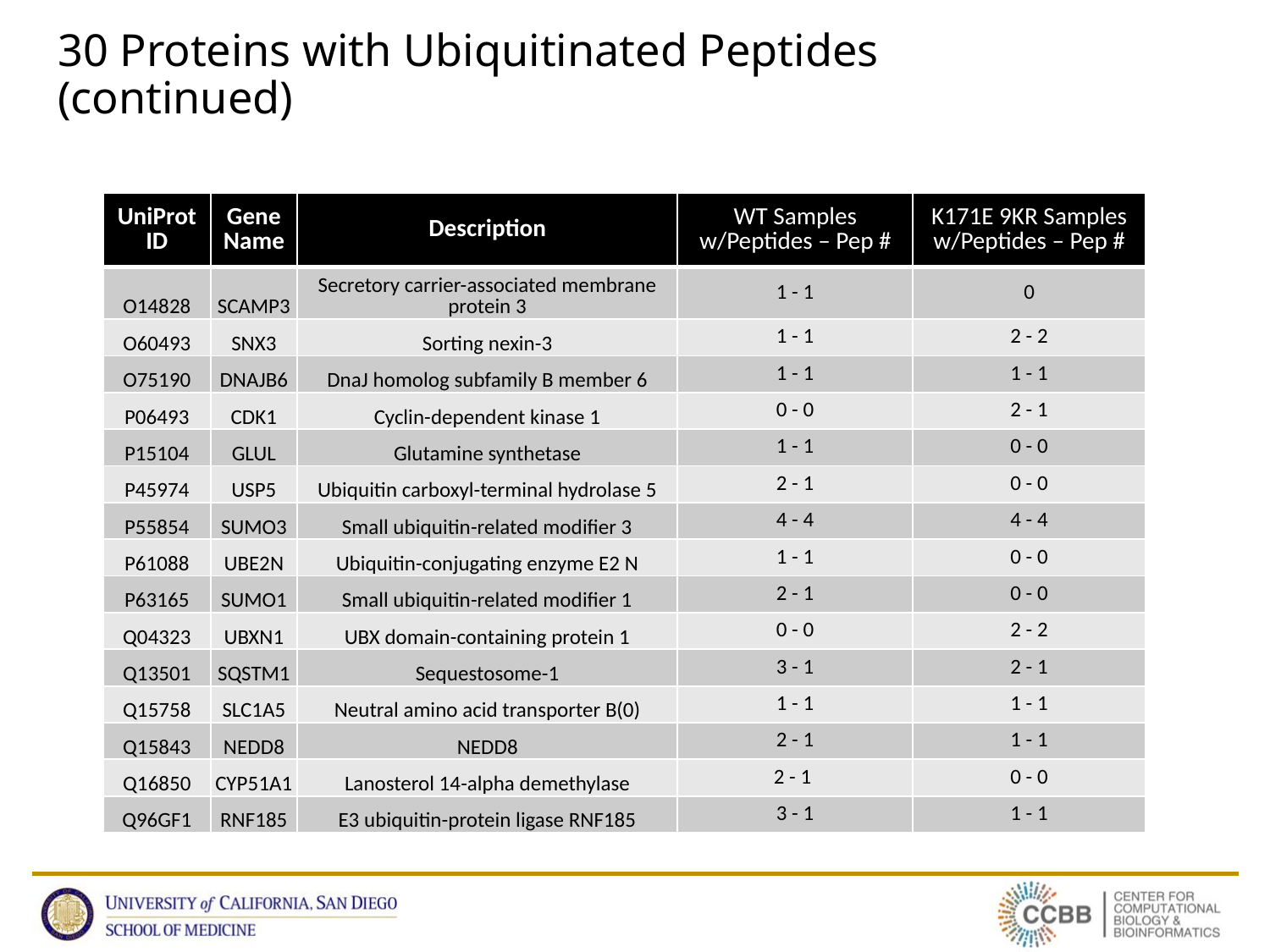

# 30 Proteins with Ubiquitinated Peptides (continued)
| UniProt ID | Gene Name | Description | WT Samples w/Peptides – Pep # | K171E 9KR Samples w/Peptides – Pep # |
| --- | --- | --- | --- | --- |
| O14828 | SCAMP3 | Secretory carrier-associated membrane protein 3 | 1 - 1 | 0 |
| O60493 | SNX3 | Sorting nexin-3 | 1 - 1 | 2 - 2 |
| O75190 | DNAJB6 | DnaJ homolog subfamily B member 6 | 1 - 1 | 1 - 1 |
| P06493 | CDK1 | Cyclin-dependent kinase 1 | 0 - 0 | 2 - 1 |
| P15104 | GLUL | Glutamine synthetase | 1 - 1 | 0 - 0 |
| P45974 | USP5 | Ubiquitin carboxyl-terminal hydrolase 5 | 2 - 1 | 0 - 0 |
| P55854 | SUMO3 | Small ubiquitin-related modifier 3 | 4 - 4 | 4 - 4 |
| P61088 | UBE2N | Ubiquitin-conjugating enzyme E2 N | 1 - 1 | 0 - 0 |
| P63165 | SUMO1 | Small ubiquitin-related modifier 1 | 2 - 1 | 0 - 0 |
| Q04323 | UBXN1 | UBX domain-containing protein 1 | 0 - 0 | 2 - 2 |
| Q13501 | SQSTM1 | Sequestosome-1 | 3 - 1 | 2 - 1 |
| Q15758 | SLC1A5 | Neutral amino acid transporter B(0) | 1 - 1 | 1 - 1 |
| Q15843 | NEDD8 | NEDD8 | 2 - 1 | 1 - 1 |
| Q16850 | CYP51A1 | Lanosterol 14-alpha demethylase | 2 - 1 | 0 - 0 |
| Q96GF1 | RNF185 | E3 ubiquitin-protein ligase RNF185 | 3 - 1 | 1 - 1 |
